# Estrogen/Esr2b signaling‐dependent, female‐biased expression of a neuropeptide Y gene in the medaka brain

**DOI:** 10.1111/jne.70279

**Published:** 2026-09-30

**Authors:** Yuki Homan, Yuji Nishiike, Mika Sato‐Tsukamoto, Daisuke Saito, Mikita Suyama, Yasuyuki Ohkawa, Fumiya Takahashi, Takashi Baba, Ken‐ichirou Morohashi, Kataaki Okubo

**Affiliations:** ^1^ Department of Aquatic Bioscience, Graduate School of Agricultural and Life Sciences The University of Tokyo Tokyo Japan; ^2^ Division of Bioinformatics, Medical Institute of Bioregulation Kyushu University Fukuoka Japan; ^3^ Division of Transcriptomics, Medical Institute of Bioregulation Kyushu University Fukuoka Japan; ^4^ Department of Molecular Biology Graduate School of Medical Sciences, Kyushu University Fukuoka Japan; ^5^ Department of Systems Life Sciences Graduate School of Systems Life Sciences, Kyushu University Fukuoka Japan; ^6^ Department of Anatomy Kurume University School of Medicine Kurume Japan; ^7^ Department of Internal Medicine Kurume University School of Medicine Kurume Japan

**Keywords:** estrogen, estrogen receptor, mating behavior, neuropeptide Y, teleost

## Abstract

A recent study showed that female medaka lacking the estrogen receptor gene *esr2b* are unreceptive to male courtship and instead court other females. In this study, we investigated the brain transcriptome of *esr2b*‐deficient females to explore the underlying mechanisms of this behavioral phenotype, and identified *npyb*, one of the two neuropeptide Y (NPY) genes in teleosts, as substantially downregulated. Follow‐up analyses revealed that *npyb* is expressed in a markedly female‐biased manner, dependent on estrogen/Esr2b signaling, in a ventral telencephalic nucleus implicated in mating behavior. These findings led us to hypothesize that *npyb* may mediate the phenotypic consequence of *esr2b* deficiency. To test this, we generated *npyb*‐deficient medaka and assessed their mating behavior; however, no notable abnormalities were observed. Given that *npya*, the other NPY gene in teleosts, is also expressed in the same brain nucleus, we considered the possibility that *npya* compensates for the loss of *npyb* function. Nevertheless, even medaka lacking both *npya* and *npyb* showed no discernible deficits in mating behavior. While the precise physiological roles of NPY in medaka remain elusive, our findings establish *npyb* as a downstream target of estrogen/Esr2b signaling and demonstrate its female‐biased expression in a behaviorally relevant brain region.

## 1 INTRODUCTION

Mating behaviors in males and females are typically innate and sex‐specific.^1^ Males often engage in elaborate courtship to attract females, while females assess these displays to decide whether to mate. In vertebrates, such behaviors are largely shaped by sex steroid hormones, including androgens, estrogens, and progestins.^2^, ^3^ These hormones, secreted by the gonads in a sex‐specific manner, act on the neural substrates of mating behavior to establish male‐ and female‐typical behavioral patterns. Their effects are primarily mediated by nuclear receptors that function as transcription factors, modulating the expression of target genes. However, despite recent advances in identifying steroid receptor–expressing neurons essential for mating behaviors, the specific downstream genes that mediate steroid receptor signaling and drive sex‐typical behavioral patterns remain largely unidentified.^4^, ^5^

We recently found that female medaka (*Oryzias latipes*) lacking *esr2b*, one of the three estrogen receptor (ESR) genes, retain normal ovarian function with an unaltered sex steroid milieu but are completely unreceptive to males and instead court other females.^6^ This finding suggests that estrogen/Esr2b signaling is essential for inducing female‐typical mating behavior and sexual preference, while suppressing those typical of males. Unlike *esr2b*, deficiency of the other two ESR genes in medaka, *esr1* and *esr2a*, does not affect female mating behavior,^7^, ^8^ underscoring the distinct role of Esr2b.

The only downstream target of estrogen/Esr2b signaling currently known to influence female mating behavior is *npba*, a gene encoding neuropeptide B. Under the control of estrogen/Esr2b signaling, *npba* is expressed in a female‐specific manner in brain nuclei implicated in teleost mating behavior, and loss of its function impairs female mating behavior.^6^, ^9^, ^10^ Nonetheless, the resulting behavioral deficits are relatively modest, implying the presence of additional crucial targets downstream of estrogen/Esr2b signaling.

In the present study, we therefore investigated the brain transcriptome of *esr2b*‐deficient females to explore additional downstream targets, which led to the identification of a gene encoding neuropeptide Y (NPY) as one of the most significantly downregulated. NPY is a 36‐amino acid polypeptide that belongs to a family of peptides including peptide YY (PYY) and pancreatic polypeptide (PP).^11^ In mammals, this peptide regulates a variety of physiological processes, including food intake, energy homeostasis, anxiety, stress resilience, and nociception.^11^, ^12^, ^13^ Its effects also extend to mating behavior: central administration of NPY suppresses mating behavior in rodents of both sexes.^14^, ^15^, ^16^ Similar effects have been reported in *Drosophila*, where the NPY homolog neuropeptide F (NPF) inhibits male mating behavior,^17^ suggesting that the role of NPY in the control of mating behavior is evolutionarily conserved. These findings led us to test here the possibility that NPY functions downstream of estrogen/Esr2b signaling to regulate mating behavior in medaka.

## 2 MATERIALS AND METHODS

### 2.1 Animals

Wild‐type d‐rR strain medaka and mutant medaka deficient in *esr2b*,^6^ *npya*, *npyb*, or both *npya* and *npyb* (generated in this study) were reared at 28°C under a 14‐h light/10‐h dark cycle. They were fed 3–4 times daily with live *Artemia nauplii* and commercial pellets (Otohime; Marubeni Nisshin Feed, Tokyo, Japan). Sexually mature adults (2–5 months of age) were used in all experiments and were randomly assigned to experimental groups. Tissue samples were consistently collected 1–3 h after lights‐on. In each analysis of the mutants, siblings raised under identical conditions were used as the comparison group to minimize genetic and environmental variability.

### 2.2 RNA sequencing (RNA‐seq)

Brains were dissected into four regions: (i) the telencephalon/olfactory bulb, (ii) the midbrain tegmentum/diencephalon, (iii) the optic tectum/optic nerves, and (iv) the cerebellum/medulla oblongata. Total RNA was isolated from each region using the RNeasy Plus Universal Mini Kit (Qiagen, Hilgen, Germany) with DNase treatment (Qiagen), followed by mRNA enrichment with the NEBNext Poly(A) mRNA Magnetic Isolation Module (New England Biolabs, Ipswich, MA). Sequencing libraries were constructed using the NEBNext Ultra II Directional RNA Library Prep with Sample Purification Beads and NEBNext Multiplex Oligo for Illumina (New England Biolabs). Paired‐end sequencing was performed on an Illumina NovaSeq 6000 System (Illumina, San Diego, CA), yielding 16–33 million reads per sample. Reads were mapped to the medaka reference genome (ASM223467) using Hisat2 (v2.1.0).^18^ Mapped reads of each sample were assembled into transcripts using StringTie (v1.3.5),^19^ and transcriptomes were merged using GffCompare (v0.10.6).^20^

Differential gene expression between genotypes was analyzed using the edgeR package (v3.24.3),^21^ with a false discovery rate (FDR) threshold of 0.05. Because the *esr2b*‐deficient line used in this study carries a Cab strain‐derived chromosomal segment encompassing the *esr2b* locus on the d‐rR strain background,^6^ differentially expressed genes (DEGs) mapped near the *esr2b* locus were excluded to avoid potential false positives arising from strain‐specific polymorphisms. Single‐exon genes were also excluded to avoid false positives. Raw mapped reads were visualized and manually inspected using Integrative Genomics Viewer (v2.4.19).^22^

### 2.3 In silico cloning and phylogenetic tree analysis

The sequence obtained by RNA‐seq (gene ID XLOC_008613, later confirmed as *npyb*) was BLAST searched against the GenBank nucleotide database to retrieve the corresponding full‐length cDNA. This cDNA sequence was further verified by identifying matching expressed sequence tag (EST) clones (e.g., clone ID olbr36n19) in the medaka EST database curated by the National BioResource Project (NBRP) Medaka (https://shigen.nig.ac.jp/medaka/). Similarly, the cDNA sequences of *npya*, *pyya*, and *pyyb* were obtained by BLAST searching the GenBank protein database using their orthologs from other species as queries. For *npya* and *pyyb*, the presence of matching EST clones in the medaka EST database (e.g., olbr31b22 and olec47l05, respectively) further supported the accuracy of the retrieved sequences.

The mature NPY polypeptide was predicted using InterPro.^23^ The deduced amino acid sequences of the medaka cDNAs/ESTs were aligned with those of their putative orthologs and paralogs from other species using ClustalW. Neighbor‐joining trees with 1000 bootstrap replicates were constructed based on these alignments using Genetyx software (Genetyx Corporation, Tokyo, Japan). The tree of NPY was rooted using human and mouse PYY sequences as outgroups, and that of PYY using human and mouse NPY. Species names and GenBank accession numbers of all sequences used are listed in Table S1.

### 2.4 Real‐time PCR

Brains were dissected into four regions, and total RNA was isolated from each region using the same protocol as for RNA‐seq. First‐strand cDNA was synthesized using the SuperScript VILO cDNA Synthesis Kit (Thermo Fisher Scientific, Waltham, MA). Real‐time PCR was performed on a LightCycler 480 System II (Roche Diagnostics, Basel, Switzerland) using the LightCycler 480 SYBR Green I Master (Roche Diagnostics) and primers listed in Table S2. Amplification specificity was verified by melting curve analysis, which yielded a single peak for each reaction. Data were normalized to *β*‐actin (*actb*; GenBank accession number NM_001104808), which has been validated as a reference gene for use in the medaka brain.^24^ Expression levels were calculated relative to the following reference groups: the telencephalon/olfactory bulb region of wild‐type males (Figures 1E, 4F, S3F, and S3G), whole brains of wild‐type females (Figures 3C, S4C, S6A, and S6B), and whole brains of females heterozygous for both *npya* and *npyb* mutations (Figure S6C).

**FIGURE 1:**
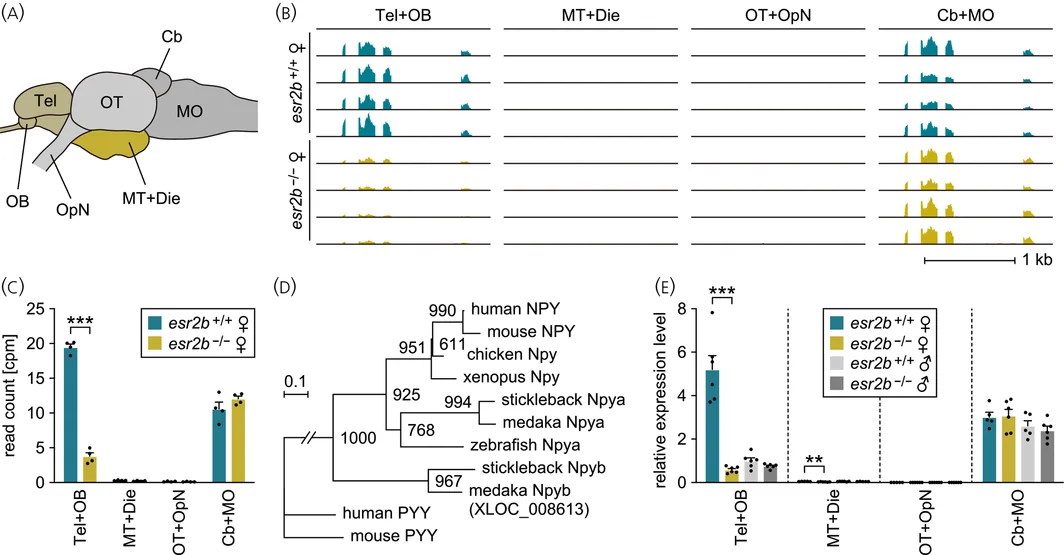
Identification of *npyb* as a downregulated gene in the brain of *esr2b*‐deficient females. (A) Lateral schematic of the medaka brain (anterior to the left) indicating the four regions dissected for RNA‐seq analysis: (i) the telencephalon/olfactory bulb (Tel + OB), (ii) the midbrain tegmentum/diencephalon (MT + Die), (iii) the optic tectum/optic nerves (OT + OpN), and (iv) the cerebellum/medulla oblongata (Cb + MO). (B) Integrative genomics viewer screenshots showing RNA‐seq reads aligned to XLOC_008613 (*npyb*) in each of the four brain regions from *esr2b* ^+/+^ and *esr2b* ^−/−^ females (*n* = 4 per genotype). (C) RNA‐seq read counts (cpm) for XLOC_008613 (*npyb*) in each brain region of *esr2b* ^+/+^ and *esr2b* ^−/−^ females (*n* = 4 per genotype). Read counts are shown in counts per million (cpm). (D) Phylogenetic tree illustrating the relationship of XLOC_008613 (medaka Npyb) to other NPY homologs. Numbers at nodes indicate bootstrap values from 1000 replicates. Scale bar: 0.1 substitutions per site. (E) Relative expression levels of XLOC_008613 (*npyb*) in each brain region of *esr2b* ^+/+^ and *esr2b* ^−/−^ females and males, measured by real‐time PCR (*n* = 5–6 per sex per genotype). Species names and GenBank accession numbers are provided in Table S1. Statistical significance was evaluated using unpaired *t*‐test with Bonferroni–Dunn correction (C) and Bonferroni's or Dunn's post hoc test following ANOVA (E). Error bars represent SEM. **p< .01, ***p< .001.

### 2.5 Single‐label in situ hybridization

DNA fragments corresponding to nucleotides 69–1034 (966 bp) of the *npya* cDNA (GenBank accession number XM_011480646), 16–451 (436 bp) of the *npyb* cDNA (XM_020710138), 759–1029 (271 bp) of the *pyya* cDNA (XM_023957886), and 72–501 (430 bp) of the *pyyb* cDNA (XM_023949591) were amplified by PCR and subcloned into the pGEM‐T Easy vector (Promega, Madison, WI). The resulting constructs were used to synthesize digoxigenin (DIG)‐labeled cRNA probes for *npya*, *npyb*, *pyya*, and *pyyb* using the DIG RNA Labeling Mix and T7 RNA polymerase (Roche Diagnostics).

The procedure for single‐label in situ hybridization followed a previously published method.^9^ Briefly, brains were fixed in 4% paraformaldehyde, embedded in paraffin, and coronally sectioned at a thickness of 10 μm. Sections were hybridized with the probes described above, followed by detection of hybridization signals using an alkaline phosphatase‐conjugated anti‐DIG antibody (RRID: AB_514497; Roche Diagnostics) and 5‐bromo‐4‐chloro‐3‐indolyl phosphate/nitro blue tetrazolium (BCIP/NBT) substrate (Roche Diagnostics). In qualitative analyses, color development was allowed to proceed overnight, whereas in quantitative analyses it was limited to 2 h for *npya* and 1.5 h for *npyb* to avoid signal saturation. Sections were then imaged using a virtual slide microscope (VS120; Olympus, Tokyo, Japan), followed by calculation of the total expression signal area in each brain region using cellSens software (Olympus). To minimize variability, all sections being compared were processed in parallel. Brain nuclei were identified based on medaka brain atlases,^25^, ^26^ aided by our own Nissl‐stained reference sections.^27^

### 2.6 Ovariectomy and hormone treatments

The procedure for ovariectomy and hormone treatments has been previously described.^28^ Briefly, females were anesthetized with 0.02% tricaine methane sulfonate (Sigma‐Aldrich, St. Louis, MO), and a small incision was made in the ventrolateral abdominal wall. The ovary was carefully removed, and the incision was closed using nylon sutures. Sham‐operated females underwent the same surgical procedure except that the ovary remained intact. Following surgery, fish were allowed to recover for 3 days in saline (0.9% (w/v) NaCl). Ovariectomized fish were subsequently exposed for 5 days to water containing 100 ng/mL of either estradiol‐17*β* (E2; the principal estrogen in vertebrates, including teleosts), 11‐ketotestosterone (11KT; the primary, non‐aromatizable androgen in teleosts), or ethanol (vehicle control). Sham‐operated fish were treated with vehicle alone. The concentrations of E2 and 11KT were selected to approximate circulating levels previously reported in medaka.^29^

### 2.7 Double‐label in situ hybridization

A DNA fragment corresponding to nucleotides 2080–3288 (1209 bp) of the *esr2b* cDNA (GenBank accession number XM_020713365) was amplified by PCR and used to generate a DIG‐labeled *esr2b* probe as described above. The above‐mentioned *npya* and *npyb* probes were labeled with either DIG or fluorescein, the latter using Fluorescein RNA Labeling Mix and T7 RNA polymerase (Roche Diagnostics).

Double‐label in situ hybridization was performed following a previously published protocol.^30^ Brains were fixed in 4% paraformaldehyde, embedded in paraffin, and coronally sectioned at 10 μm thickness. These sections were hybridized simultaneously with fluorescein‐labeled *npyb* and DIG‐labeled *esr2b* probes. Alternatively, brains were embedded in 5% agarose (Type IX‐A; Sigma‐Aldrich) supplemented with 20% sucrose and coronally cryosectioned at 20 μm thickness. These sections were hybridized simultaneously with fluorescein‐labeled *npya* and DIG‐labeled *npyb* probes. DIG‐labeled signals were detected with an alkaline phosphatase‐conjugated anti‐DIG antibody (RRID: AB_514497; Roche Diagnostics) and visualized with Fast Red (Roche Diagnostics), while fluorescein‐labeled signals were detected with a horseradish peroxidase‐conjugated anti‐fluorescein antibody (RRID: AB_2737388; PerkinElmer, Waltham, MA) and visualized using the TSA Plus Fluorescein System (Akoya Biosciences, Marlborough, MA). Sections were counterstained with 4′,6‐diamidino‐2‐phenylindole (DAPI) and imaged using a confocal laser‐scanning microscope (TCS SP8; Leica Microsystems, Wetzlar, Germany). Excitation/emission wavelengths were set at 405/410–480 nm for DAPI, 488/495–545 nm for fluorescein, and 552/620–700 nm for Fast Red.

To quantify the coexpression of *npyb* and *esr2b*, confocal images of the Vs/Vp were manually examined. For each of five female fish, 50 labeled neurons (10 neurons randomly selected from each of five sections) were analyzed, and the proportions of double‐positive neurons among *npyb*‐positive and *esr2b*‐positive neurons were calculated.

### 2.8 Transcriptional activity assay

A medaka fosmid clone (clone ID GOLWFno680_m05) containing the *npyb* locus was obtained from the NBRP Medaka. The *npyb* gene and flanking regions were analyzed for the presence of potential canonical bipartite estrogen‐responsive element (ERE)‐like sequences using Jaspar (version 5.0_alpha) with default settings.

Transcriptional activity assay was performed as described previously.^6^ Briefly, a 2973‐bp genomic fragment upstream of the first methionine codon of *npyb* (containing 2755 bp of the 5′‐flanking region, the entire exon 1 and intron 1, and 20 bp of exon 2) and a 3000‐bp fragment downstream of the stop codon of *npyb* (containing the entire 3′‐untranslated region and 2539 bp of the 3′‐flanking region) were PCR‐amplified from the fosmid clone. These fragments were ligated into the NheI and XbaI sites, respectively, of the pGL4.10 luciferase reporter vector (Promega). The resulting luciferase reporter construct was transiently transfected into COS‐7 cells along with an expression plasmid encoding medaka Esr1 (GenBank accession number XM_020714493), Esr2a (NM_001104702), or Esr2b (XM_020713365), and the internal control vector pGL4.74 (Promega), using Lipofectamine LTX (Thermo Fisher Scientific). At 6 h post‐transfection, cells were treated with E2 at concentrations of 0, 10^−9^, 10^−8^, 10^−7^, 10^−6^, and 10^−5^ M for 18 h. Cell lysates were then assayed for luciferase activity using the Dual‐Glo Luciferase Assay System (Promega) on a GloMax Explorer Multimode Microplate Reader (Promega). Assays were performed in duplicate and repeated independently three times. Data were expressed relative to unstimulated control cells.

### 2.9 Generation of mutant medaka

*npya*‐ and *npyb*‐deficient medaka were generated using clustered regularly interspaced short palindromic repeats (CRISPR)/CRISPR‐associated protein 9 (Cas9)‐mediated genome editing. Two CRISPR RNAs (crRNAs) were designed for *npya* and one for *npyb*, each targeting the mature polypeptide‐coding region. The crRNAs and trans‐activating crRNA (tracrRNA) were synthesized by Fasmac (Kanagawa, Japan) and co‐injected with Cas9 protein (Fasmac) into the cytoplasm of one‐cell stage embryos.

Injected fish were raised to adulthood and outcrossed with wild‐type fish. Their progeny were screened for target site mutations using a T7 endonuclease I assay,^31^ followed by direct sequencing. For each gene, two founders were identified that transmitted deletions causing frameshifts and premature translation termination. Progeny from each founder were intercrossed to establish two independent mutant lines per gene. These lines were maintained by intercrossing heterozygotes, yielding both wild‐type and homozygous siblings for experiments. Genotyping was performed by PCR amplification of the target locus, followed by agarose gel electrophoresis for *npya*‐deficient lines or high‐resolution melting analysis for *npyb*‐deficient lines, using the primers and probe listed in Table S2. Double‐deficient (*npya*/*npyb*) fish were generated by intercrossing the respective single‐deficient lines. For experiments using these fish, sibling fish that were wild‐type or heterozygous for both genes were used as controls.

### 2.10 Mating behavior test

The mating behavior test was conducted as previously described.^10^ On the day before testing, each focal fish was placed in a 2‐L rectangular tank with a wild‐type stimulus fish, separated by a transparent perforated partition. 1 h after lights‐on on the following day, the partition was removed, allowing the fish to interact freely for 30 min. In the female–female mating behavior test, the interaction period was instead extended to 2 h, and stimulus fish were marked with small notches in the caudal fin to enable individual identification. All behaviors were recorded using a digital video camera (iVIS HF S11/S21, Canon, Tokyo, Japan; Everio GZ‐G5, Jvckenwood, Kanagawa, Japan; or HC‐W870M, Panasonic, Osaka, Japan). From the video recordings, the following behavioral parameters were measured blind to the genotype of the fish: the latencies to the first following, courtship display, and wrapping by the male, and to spawning, as well as the number of courtship displays before spawning and the number of wrapping attempts rejected by the female.

### 2.11 Statistical analysis

Quantitative results were presented as mean ± standard error of the mean (SEM), and individual measurements were plotted on the graphs to visualize the data distribution. Time‐series behavioral data were analyzed using Kaplan–Meier plots, which allow for the inclusion of individuals that did not exhibit the behavior within the observation period.^32^

Statistical calculations were carried out using GraphPad Prism (GraphPad Software, San Diego, CA). For comparisons between two groups, unpaired two‐tailed Student's *t‐*tests were used, with Welch's correction applied when the *F* test indicated unequal variances. For multiple pairwise comparisons, significance thresholds were adjusted using the Bonferroni–Dunn method. Comparisons among three or more groups were conducted using one‐way analysis of variance (ANOVA), followed by either Bonferroni's post hoc test for intergroup comparisons or Dunnett's post hoc test for comparisons with a control group. When the Brown–Forsythe test detected heterogeneity of variance, the non‐parametric Kruskal–Wallis test was applied instead, followed by Dunn's post hoc test. Kaplan–Meier curves were compared using the Gehan–Breslow–Wilcoxon test. All measurements were included in the statistical analyses without excluding outliers.

## 3 RESULTS

### 3.1 Identification of *npyb* as a downregulated gene in the brain of *esr2b*‐deficient females

To explore genes that mediate the effects of estrogen/Esr2b signaling on female mating behavior, we performed RNA‐seq on brains of *esr2b*‐deficient females, which were dissected into four regions (Figure 1A). Differential expression analysis identified 14 DEGs between *esr2b* ^+/+^ and *esr2b* ^−/−^ females (Table S3). Among these, XLOC_024810 and XLOC_008613 exhibited the most significant differences (FDR = 3.36 × 10^−15^ and 2.71 × 10^−11^, respectively). Because XLOC_024810 was annotated as *npba*, which has been previously linked to female mating behavior,^10^ we focused here on XLOC_008613. Inspection of mapped reads revealed that XLOC_008613 expression was markedly reduced in the telencephalon/olfactory bulb region of *esr2b* ^−/−^ females, to 18% of the wild‐type level (*p*< .001), whereas expression in the other regions remained low and unaltered (Figure 1B,C). In silico cloning showed that XLOC_008613 matched a GenBank entry annotated as a predicted member of the NPY family (XM_020710138/XP_020565797), recently referred to as *npyb*.^33^ Phylogenetic analysis confirmed that this gene encodes the medaka ortholog of Npyb found in other teleost species (Figure 1D).

To validate the RNA‐seq results, we quantified *npyb* expression in the same four brain regions using real‐time PCR. To evaluate potential sex differences, *esr2b* ^+/+^ and *esr2b* ^−/−^ males were also included in this analysis. As expected, *npyb* expression in the telencephalon/olfactory bulb region of *esr2b* ^−/−^ females was significantly reduced to 8.8% of that in wild‐type females (*p*< .001) (Figure 1E). Notably, *npyb* expression in this region was strongly female‐biased: levels in *esr2b* ^+^/^+^ males were as low as those in *esr2b* ^−/−^ females, and loss of *esr2b* had no additional effect in males. A significant reduction was also detected in the midbrain tegmentum/diencephalon region of *esr2b* ^−/−^ females, although baseline expression in this region was minimal (*p* = .006 vs. *esr2b* ^+/+^ females) (Figure 1E).

Together, these results demonstrate that *npyb* expression in the telencephalon and/or olfactory bulb is dependent on Esr2b and shows a strong female bias.

### 3.2 Estrogen/Esr2b signaling‐dependent, female‐biased expression of *npyb* in the ventral telencephalon

We next performed in situ hybridization to identify the brain nucleus responsible for the reduced *npyb* expression in *esr2b*‐deficient females and its female‐biased expression. *npyb* expression was detected in the Vs/Vp of the ventral telencephalon, the PMp of the preoptic area, and the gc of the brainstem (Figure 2A,B; abbreviations for medaka brain nuclei are provided in Table S4). Among these, *npyb* expression in the Vs/Vp was markedly reduced in *esr2b* ^−/−^ females, to 16% of that in *esr2b* ^+/+^ females (*p*< .001) (Figure 2C,D). The Vs/Vp also showed female‐biased expression, with significantly higher levels in *esr2b* ^+/+^ females than in *esr2b* ^+/+^ males (*p* = .003). Additionally, expression in *esr2b* ^−/−^ males was significantly lower than in *esr2b* ^+/+^ males (*p* = .026), indicating that the effect of *esr2b* deficiency extends to males. In contrast, *npyb* expression in the PMp was detected almost exclusively in males (Figure 2C); however, due to low overall expression levels and high individual variability, no statistically significant sex differences or effects of *esr2b* deficiency were observed (Figure 2D). In the gc, *npyb* was broadly expressed along the rostrocaudal axis and this expression extended into the spinal cord, precluding accurate quantification. However, RNA‐seq and real‐time PCR analyses of the cerebellum/medulla oblongata region, including the gc, revealed no effect of *esr2b* deficiency or sex, suggesting that *npyb* expression in the gc is neither *esr2b*‐ nor sex‐dependent. Together, these results demonstrate that the Vs/Vp of the ventral telencephalon is the source of both the *esr2b* deficiency‐induced downregulation and the female‐biased expression of *npyb*.

**FIGURE 2:**
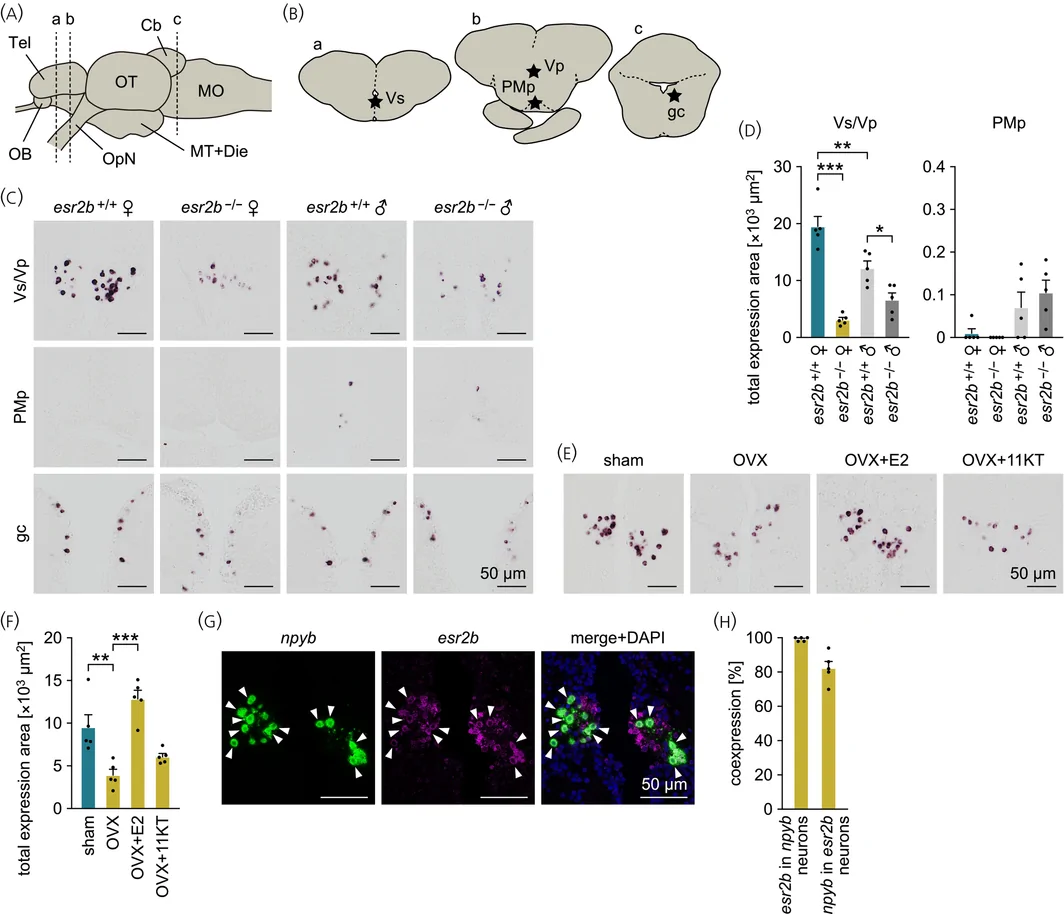
Estrogen/Esr2b signaling‐dependent, female‐biased expression of *npyb* in the ventral telencephalon. (A) Lateral schematic of the medaka brain (anterior to the left) indicating section planes shown in panel (B). (B) Coronal brain sections highlighting nuclei with detectable *npyb* expression (indicated by stars). (C) Representative images of *npyb* expression in each brain nucleus of *esr2b* ^+/+^ and *esr2b* ^−/−^ females and males. (D) Total area of *npyb* expression in the Vs/Vp and PMp of *esr2b* ^+/+^ and *esr2b* ^−/−^ females and males (*n* = 5 per sex per genotype). (E) Representative images of *npyb* expression in the Vs/Vp of sham‐operated (sham) females and ovariectomized females that were exposed to vehicle alone (OVX), estradiol‐17β (OVX + E2), or 11‐ketotestosterone (OVX + 11KT). (F) Total area of *npyb* expression in the Vs/Vp of sham, OVX, OVX + E2, and OVX + 11KT females (*n* = 5 per group). (G) Expression of *esr2b* in *npyb*‐expressing neurons within the female Vs/Vp. Left and middle panels show *npyb* (green) and *esr2b* (magenta) expression, respectively, in the same section; right panel shows the merged image with nuclear counterstain (blue). Arrowheads indicate representative *npyb*‐expressing neurons that coexpress *esr2b*. Scale bars: 50 μm. (H) Percentages of *npyb*‐expressing neurons coexpressing *esr2b* and of *esr2b*‐expressing neurons coexpressing *npyb* in the female Vs/Vp (*n* = 5). See Table S4 for abbreviations of brain regions and nuclei. Statistical significance was evaluated using Bonferroni's post hoc test following ANOVA (D), (F). Error bars represent SEM. *p< .05, **p< .01, ***p< .001.

Based on these results, we inferred that ovarian‐derived estrogens, acting via brain Esr2b, may shape the observed *npyb* expression pattern in the Vs/Vp. To test this, we first analyzed *npyb* expression in ovariectomized females treated with E2 or 11KT. In situ hybridization revealed that ovariectomy significantly reduced *npyb* expression in the Vs/Vp (*p* = .002), and this reduction was fully restored by E2 treatment (*p*< .001), suggesting dependence on ovarian‐derived estrogens. In contrast, 11KT treatment had no such effect (Figure 2E,F). Next, to determine whether estrogens can directly act on *npyb*‐expressing neurons in the Vs/Vp, we performed double‐label in situ hybridization for *npyb* and *esr2b* (Figure 2G). Quantitative analysis showed that 99% of *npyb*‐expressing neurons also expressed *esr2b*, whereas 82% of *esr2b*‐expressing neurons expressed *npyb* (Figure 2H), suggesting direct estrogen action on these cells.

To further assess the potential for direct estrogenic regulation, we searched for EREs in the medaka *npyb* locus and identified two canonical bipartite ERE‐like sequences in the upstream flanking region (positions −2307 and −1974 relative to the transcription start site) and one in the downstream flanking region (position +2094 relative to the transcription start site) (Figure S1A). Based on this finding, we investigated whether estrogens can directly stimulate *npyb* transcription by conducting a transcriptional activity assay using a luciferase reporter construct containing these flanking regions. However, no estrogen‐dependent induction of luciferase activity was observed with any Esr subtype (Figure S1B). Thus, these results provide no evidence for direct transcriptional regulation of *npyb* by estrogen signaling.

Overall, the results suggest that ovarian‐derived estrogens act on *npyb*‐expressing neurons in the Vs/Vp through Esr2b, thereby driving the female‐biased *npyb* expression that is diminished in the absence of *esr2b*.

### 3.3 No detectable deficits in mating behavior, growth, or maturation in *npyb* mutants

In view of the preceding results and the established role of the Vs/Vp in teleost mating behavior,^34^ *npyb* emerged as a strong candidate effector gene downstream of estrogen/Esr2b signaling in regulating female mating behavior. To examine this possibility, we generated *npyb*‐deficient medaka using the CRISPR/Cas9 system. To ensure reproducibility of subsequent phenotypic analyses, two independent *npyb*‐deficient lines were established (Δ5 and Δ29) and used for further analyses (Figure 3A). Both the Δ5 and Δ29 alleles carried frameshift mutations that disrupted most of the mature Npyb polypeptide (Figure 3B). Consistent with the introduced mutation, real‐time PCR showed that *npyb* transcript levels were significantly reduced in the brains of *npyb* ^−/−^ females (*p*< .001), likely due to nonsense‐mediated mRNA decay (Figure 3C). Given the established role of NPY in metabolic regulation,^11^, ^12^, ^13^ we first assessed body length, body weight, gonadal weight, and gonad/body weight ratio before examining the effects of *npyb* deficiency on behavior. These analyses revealed no significant differences between *npyb* ^−/−^ fish and wild‐type siblings of either sex in both the Δ5 and Δ29 lines (Δ5: Figure 3D–G; Δ29: Figure S2A–D), indicating that somatic growth and gonadal maturation were unaffected.

**FIGURE 3:**
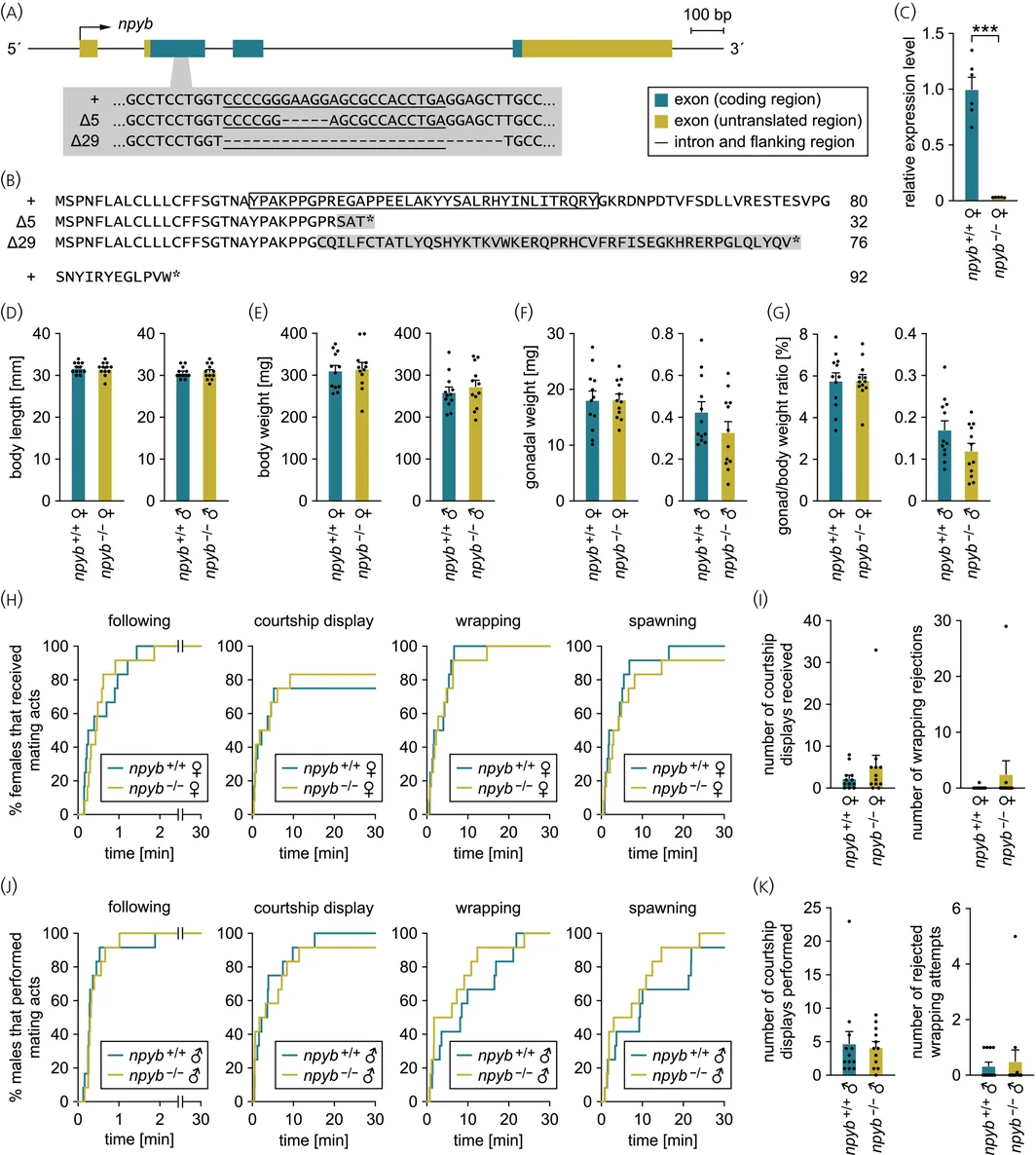
No detectable deficits in mating behavior, growth, or maturation in *npyb* mutants (Δ5 line). (A) Schematic of the *npyb* locus showing the CRISPR RNA target site, which is enlarged to depict the nucleotide sequences of the wild‐type (+) and mutant (Δ5 and Δ29) alleles. The CRISPR RNA target sequence is underlined, and deleted bases are indicated by dashes. (B) Comparison of the deduced precursor proteins of wild‐type (+) and mutant (Δ5 and Δ29) Npyb. The mature Npyb polypeptide is boxed. Altered sequences resulting from frameshifts are shaded gray. Asterisks denote stop codons. (C) Relative transcript levels of *npyb* in the brains of *npyb* ^+/+^ and *npyb* ^−/−^ females of the Δ5 line, measured by real‐time PCR (*n* = 5–6 per genotype). (D)–(G) Body length (D), body weight (E), gonadal weight (F), and gonad/body weight ratio (G) of *npyb* ^+/+^ and *npyb* ^−/−^ females and males of the Δ5 line (*n* = 12 per sex per genotype). (H) Latency of *npyb* ^+/+^ and *npyb* ^−/−^ females of the Δ5 line (*n* = 12 per genotype) to receive each mating act (following, courtship display, and wrapping) from the stimulus male and to spawn. (I) Number of courtship displays these females received from the stimulus male and wrapping attempts they rejected. (J) Latency of *npyb* ^+/+^ and *npyb* ^−/−^ males of the Δ5 line (*n* = 12 per genotype) to initiate each mating act toward the stimulus female and to spawn. (K) Number of courtship displays performed by these males and wrapping attempts rejected by the stimulus female. Statistical significance was evaluated using unpaired *t*‐test, with Welch's correction where appropriate (C)–(G), (I), (K), and Gehan–Breslow–Wilcoxon test (H), (J). Error bars represent SEM. ***p< .001.

We then assessed the impact of *npyb* deficiency on mating behavior. The mating behavior of medaka follows a well‐documented, stereotyped sequence of actions.^10^, ^35^ The sequence begins with the male approaching the female and closely following her. This is followed by characteristic courtship displays, in which the male rapidly swims in a circle in front of the female. If the female is receptive, the male proceeds to clasp her with his dorsal and anal fins (a behavior called wrapping) and the pair then quiver in synchrony, culminating in spawning. If the female is unreceptive, she either adopts a rejection posture by lifting her head or quickly swims away from the male. Behavior tests of *npyb* ^−/−^ females revealed that, in both the Δ5 and Δ29 lines, their latencies to be followed, courted, and wrapped by stimulus males, and to spawn, were all comparable to those of *npyb* ^+/+^ females. In addition, *npyb* ^−/−^ females received courtship displays and rejected wrapping attempts at rates comparable to those of *npyb* ^+/+^ females (Δ5: Figure 3H,I; Δ29: Figure S2E,F). Given that estrogen/Esr2b signaling suppresses male‐typical mating behavior and sexual preference,^6^ we additionally investigated whether *npyb* mediates this function by testing *npyb* ^−/−^ females for courtship toward stimulus females. However, in both the Δ5 and Δ29 lines, none of the 12 *npyb* ^−/−^ females examined courted stimulus females. We also examined the mating behavior of *npyb* ^−/−^ males as a reference. In both the Δ5 and Δ29 lines, none of the behavioral measures in these males differed significantly from those of *npyb* ^+/+^ males (Δ5: Figure 3J,K; Δ29: Figure S2G,H). Collectively, constitutive *npyb* deficiency resulted in no detectable deficits in mating behavior in either sex.

### 3.4 Spatial distribution of *npya* expression in the brain of wild‐type and *esr2b*‐deficient males and females

A straightforward interpretation of these results is that *npyb* is dispensable for mating behavior in medaka. However, since teleosts, including medaka, possess the paralog of *npyb*, *npya* (Figure 1D), it remains possible that *npya* functionally compensates for the loss of *npyb*, thereby masking its behavioral role. Accordingly, we tested the hypothesis that functional redundancy between these genes might conceal the behavioral phenotype of *npyb*‐deficient fish. We first identified medaka *npya* (GenBank accession numbers XM_011480646/XP_011478948) by in silico cloning and analyzed its expression in the brain using in situ hybridization. Although *npya* was not listed among the DEGs identified by RNA‐seq, we still considered it relevant to examine its link with estrogen/Esr2b signaling, given its potential role in mating behavior. Therefore, brains from both wild‐type and *esr2b*‐deficient fish were included in this analysis. The results showed that *npya* is expressed in the following brain nuclei: the Vl and Vs/Vp of the ventral telencephalon; the PMp of the preoptic area; the NVT/NRL and NPPv of the hypothalamus; and the PGZ3 of the optic tectum (Figure 4A–C; abbreviations for medaka brain nuclei are provided in Table S4). Despite its low level, the expression of *npya* in the Vs/Vp, where *npyb* is regulated by estrogen/Esr2b signaling in a sex‐dependent manner, raises the possibility that *npya* compensates for the loss of *npyb* function in this nucleus. To further assess this, we quantified *npya* expression in the Vs/Vp. However, no significant differences were observed between *esr2b* ^+/+^ and *esr2b* ^−/−^ genotypes or between sexes (Figure 4D), indicating that, unlike *npyb*, *npya* expression is not under the control of estrogen signaling. Although not quantitatively assessed, visual inspection suggested no apparent differences in *npya* expression between genotypes or sexes in other nuclei as well (Figure 4C). The expression of both *npya* and *npyb* in the Vs/Vp prompted us to examine whether they are coexpressed in the same neurons. Double‐label in situ hybridization revealed that neurons expressing *npya* and *npyb* were located in the ventral and dorsal parts of the Vs/Vp, respectively, with no colocalization detected (Figure 4E). To complement these in situ hybridization data and provide a comprehensive, quantitative assessment of *npya* expression across broader brain regions, we performed real‐time PCR analysis using the same four brain regions as examined for *npyb*. Although a small but statistically significant difference in *npya* expression was detected between *esr2b* ^+/+^ and *esr2b* ^−/−^ females in the midbrain tegmentum/diencephalon (*p* = .017), no notable differences were observed between the two genotypes in the other brain regions (Figure 4F). In the optic tectum/optic nerves, expression was male‐biased in both genotypes (*p*< .001) (Figure 4F). Overall, these results indicate that, unlike *npyb*, *npya* expression is largely independent of Esr2b signaling in the medaka brain.

**FIGURE 4:**
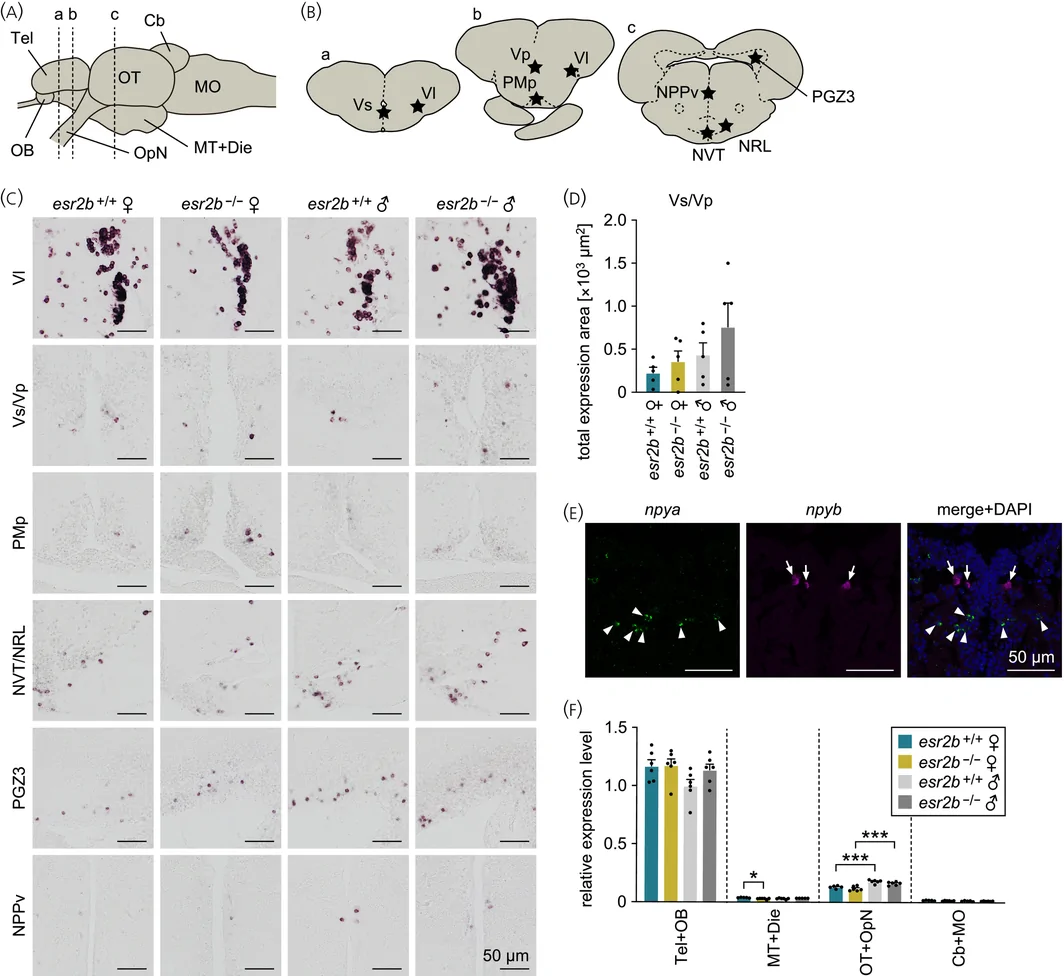
Spatial distribution of *npya* expression in the brain of wild‐type and *esr2b*‐deficient males and females. (A) Lateral schematic of the medaka brain (anterior to the left) indicating section planes shown in panel (B). (B) Coronal brain sections highlighting nuclei with detectable *npya* expression (indicated by stars). (C) Representative images of *npya* expression in each nucleus of *esr2b* ^+/+^ and *esr2b* ^−/−^ females and males. (D) Total area of *npya* expression in the Vs/Vp of *esr2b* ^+/+^ and *esr2b* ^−/−^ females and males (*n* = 5 per sex per genotype). (E) Distribution of *npya*‐ and *npyb*‐expressing neurons in the female Vs/Vp. Left and middle panels show *npya* (green) and *npyb* (magenta) expression, respectively, in the same section; right panel shows the merged image with nuclear counterstain (blue). Arrowheads and arrows denote *npya*‐ and *npyb*‐expressing neurons, respectively. Scale bars: 50 μm. (F) Relative expression levels of *npya* in each brain region of *esr2b* ^+/+^ and *esr2b* ^−/−^ females and males, measured by real‐time PCR (*n* = 5–6 per sex per genotype). See Table S4 for abbreviations of brain regions and nuclei. Statistical significance was evaluated using Bonferroni's post hoc test following ANOVA (D), (F). Error bars represent SEM. *p< .05, ***p< .001.

Another member of the NPY peptide family, PYY, shares receptors with NPY.^11^ This raised the possibility that PYY might compensate for the loss of *npyb* function. To investigate this, we first identified the relevant genes in medaka through in silico cloning. Although teleosts generally possess two paralogs of PYY, as is the case with NPY, an earlier study suggested that medaka had lost *pyyb* during evolution.^36^ Our database search and phylogenetic analysis, however, revealed that medaka retains both *pyya* (GenBank accession numbers XM_023957886/XP_02381365) and *pyyb* (XM_023949591/XP_023805359), consistent with other teleosts (Figure S3A). We then examined where these genes are expressed in the brain using in situ hybridization. Neither *pyya* nor *pyyb* expression was detected in the Vs/Vp. Instead, *pyya* was expressed in the Dm and Dl of the dorsal telencephalon, the Vl of the ventral telencephalon, the VM of the thalamus, the DT of the midbrain tegmentum, and the gc of the brainstem (Figure S3B–D). *pyyb* was expressed in the Flt of the ventral telencephalon, the Pbl of the preoptic area, the NVT and NAT of the hypothalamus, the DT of the midbrain tegmentum, and the gc of the brainstem (Figure S3B,C,E). No apparent sex differences in expression were noted in any of these nuclei. To complement the qualitative observations and quantitatively verify whether these PYY genes are regulated by estrogen/Esr2b signaling, we performed real‐time PCR analysis across the four brain regions. No significant differences in *pyya* or *pyyb* expression were detected between *esr2b* ^+/+^ and *esr2b* ^−/−^ genotypes or between sexes in any region (Figure S3F,G). These findings indicate that, similar to *npya*, neither *pyya* nor *pyyb* is under the regulatory control of estrogen/Esr2b signaling.

The findings together indicate that *npya*, like *npyb*, is expressed in the Vs/Vp and may compensate for the loss of *npyb* function in this nucleus, although coexpression of these genes in the same neurons was not observed. In contrast, *pyya* and *pyyb*, which are not expressed in the Vs/Vp, are unlikely to play such a role.

### 3.5 No detectable deficits in mating behavior, growth, or maturation in *npya*/*npyb* double mutants

To test whether the preserved mating behavior in *npyb*‐deficient medaka is due to functional compensation by *npya*, we generated fish deficient in both *npya* and *npyb* and examined their phenotypes. We first established *npya*‐deficient lines using the CRISPR/Cas9 system. Two independent mutant lines (Δ50 and Δ53) were generated to ensure reproducibility of the results (Figure S4A). Both the Δ50 and Δ53 alleles carried frameshift mutations that eliminated the mature Npya polypeptide (Figure S4B). Real‐time PCR showed that *npya* transcript levels were significantly reduced in the brains of *npya* ^−/−^ females (*p*< .001), likely due to nonsense‐mediated mRNA decay (Figure S4C). Male and female mutants of both lines exhibited normal body length, body weight, gonadal weight, and gonad/body weight ratio, as was the case in *npyb*‐deficient fish (Δ50: Figure S4D–G; Δ53: Figure S5A–D). Mating behavior was also unaffected in both sexes (Δ50: Figure S4H–K; Δ53: Figure S5E–H).

We next crossed the *npya*‐ and *npyb*‐deficient lines to generate double‐deficient fish. Despite the known involvement of NPY in metabolic regulation,^11^, ^12^, ^13^ these fish showed no overt abnormalities in body length, body weight, gonadal weight, or gonad/body weight ratio, except for a slight decrease in gonad/body weight ratio in *npya* ^−/−^/*npyb* ^−/−^ females relative to wild‐type siblings (*p* = .038) (Figure 5A–D). Behavioral tests revealed that *npya* ^−/−^/*npyb* ^−/−^ females were followed, courted, and wrapped by stimulus males and subsequently spawned, with latencies comparable to those of wild‐type females (Figure 5E). They also received courtship displays and rejected wrapping attempts at similar frequencies (Figure 5F). When tested for male‐typical mating behavior, none of the 12 *npya* ^−/−^/*npyb* ^−/−^ fish exhibited courtship toward stimulus females. Similarly, *npya* ^−/−^/*npyb* ^−/−^ males showed behavioral patterns indistinguishable from those of wild‐type males (Figure 5G,H).

**FIGURE 5:**
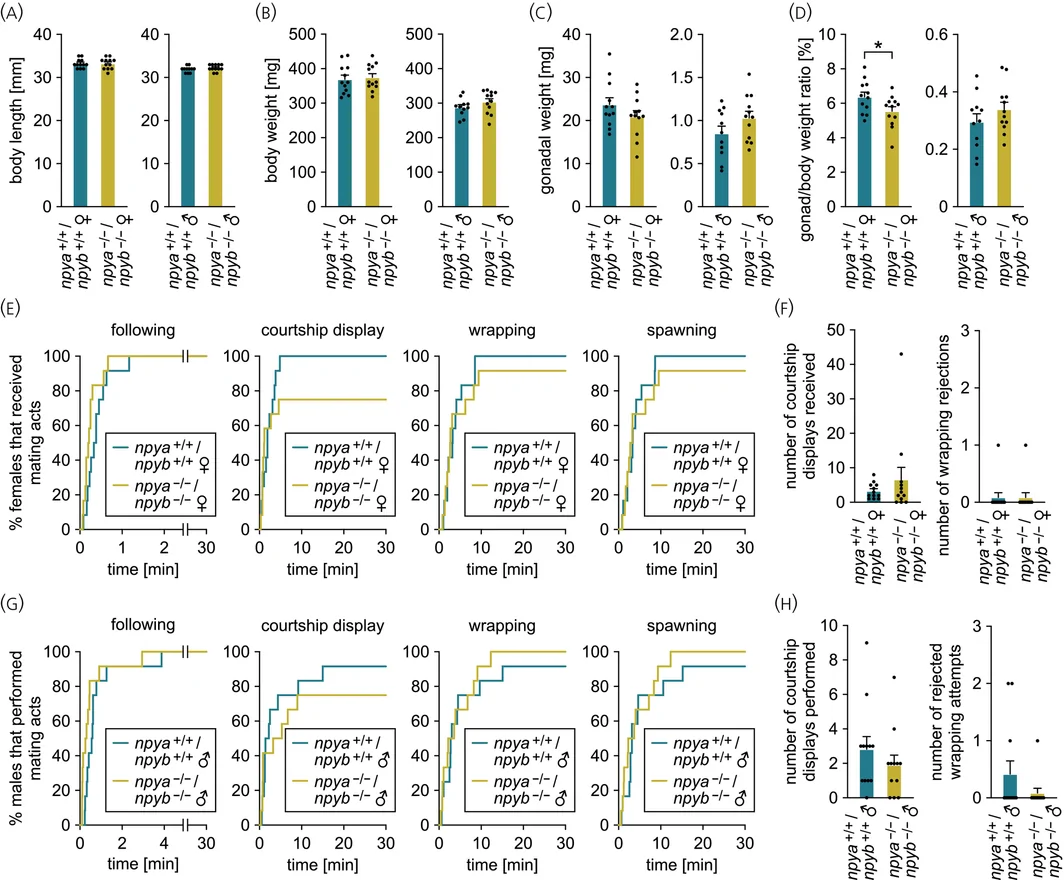
No detectable deficits in mating behavior, growth, or maturation in *npya*/*npyb* double mutants. (A)–(D) Body length (A), body weight (B), gonadal weight (C), and gonad/body weight ratio (D) of *npya* ^+/+^/*npyb* ^+/+^ and *npya* ^−/−^/*npyb* ^−/−^ females and males (*n* = 12 per sex per genotype except for *npya* ^+/+^/*npyb* ^+/+^ males, where *n* = 11). (E) Latency of *npya* ^+/+^/*npyb* ^+/+^ and *npya* ^−/−^/*npyb* ^−/−^ females (*n* = 12 per genotype) to receive each mating act (following, courtship display, and wrapping) from the stimulus male and to spawn. (F) Number of courtship displays these females received from the stimulus male and wrapping attempts they rejected. (G) Latency of *npya* ^+/+^/*npyb* ^+/+^ and *npya* ^−/−^/*npyb* ^−/−^ males (*n* = 12 per genotype) to initiate each mating act toward the stimulus female and to spawn. (H) Number of courtship displays performed by these males and wrapping attempts rejected by the stimulus female. Statistical significance was evaluated using unpaired *t*‐test, with Welch's correction where appropriate (A)–(D), (F), (H), and Gehan–Breslow–Wilcoxon test (E), (G). Error bars represent SEM. *p< .05.

To examine potential compensatory changes within the NPY/PYY system, we quantified the expression of other family members in the brains of *npyb*‐deficient, *npya*‐deficient, and *npya/npyb* double‐deficient females. Real‐time PCR revealed no significant changes in the expression of *npya*, *npyb*, *pyya*, or *pyyb* in any of these mutant lines (Figure S6). These results exclude the possibility of compensatory transcriptional upregulation among these related peptide genes.

In summary, even the simultaneous deficiency of *npya* and *npyb* resulted in no detectable deficits in mating behavior in either sex. Furthermore, no overt impacts on somatic growth or gonadal maturation were observed.

## 4 DISCUSSION

The present study identified *npyb* as markedly downregulated in the brain of *esr2b*‐deficient female medaka, which are unreceptive to male courtship and instead court other females.^6^ This downregulation was localized to the Vs/Vp, a brain nucleus known to regulate mating behavior in teleosts and to constitute a conserved node of the social decision‐making network across vertebrates,^34^, ^37^ where *npyb* expression was found to be strikingly female‐biased. These findings, together with the established role of NPY in reproductive behavior across rodents and flies,^14^, ^15^, ^16^, ^17^ led us to hypothesize that reduced *npyb* expression in the Vs/Vp might underlie the atypical behavior of *esr2b*‐deficient females. However, contrary to our expectations, females lacking *npyb* mated normally with males and did not court other females. This result led us to further investigate whether *npya*, a paralog of *npyb* that is also expressed in the Vs/Vp, might compensate for the loss of *npyb* function. Yet, females lacking both *npya* and *npyb* still mated normally. Males lacking both genes likewise showed no mating deficits. Thus, our findings suggest that NPY signaling is not essentially required for mating behavior in medaka. Although NPY‐mediated regulation of mating behavior appears evolutionarily ancient,^17^ its role may not be broadly conserved across all taxa.

Several methodological factors, however, could account for the lack of observable behavioral phenotypes in our study. First, because we utilized constitutive mutants, developmental compensation, such as changes in receptor sensitivity or functional substitution by other neural systems, might have occurred to mask the behavioral consequences of NPY deficiency. Acute pharmacological manipulations, such as administration of NPY peptides or receptor antagonists in adult animals, could help bypass potential developmental compensation, although established protocols and selective tools for this approach in medaka remain limited. Second, while our assays captured the primary components of medaka mating behavior, we cannot fully exclude the possibility that more detailed behavioral tracking, capable of evaluating fine‐scale variations in courtship vigor, male persistence, or female receptivity thresholds, might reveal subtle abnormalities. Nevertheless, given that the loss of NPY signaling does not cause overt deficits in mating behavior under the conditions tested, the primary function of this pathway in the Vs/Vp may lie elsewhere.

What, then, is the physiological role of the estrogen/Esr2b signaling‐dependent, female‐biased expression of *npyb* in the Vs/Vp? *npyb*‐expressing neurons reside in the dorsal Vs/Vp, which has been proposed to be homologous to the rodent central amygdala (whereas the ventral Vs/Vp, which contains *npya*‐expressing neurons, is considered to correspond to the bed nucleus of the stria terminalis).^38^ In rodents, NPY synthesized in the amygdala is well known for its anxiolytic effects.^39^, ^40^ Zebrafish (*Danio rerio*) lacking *npya*, their sole NPY paralog due to the loss of *npyb*, exhibit anxiety‐like behavior,^41^ suggesting a conserved anxiolytic role for NPY in teleosts. However, since *npya* is not expressed in the Vs/Vp of zebrafish,^42^, ^43^, ^44^, ^45^ its anxiolytic function is likely mediated through other brain regions. These observations suggest that NPY expression in the Vs/Vp is not essential for anxiolytic regulation in teleosts. Further investigation is needed to clarify its specific physiological significance.

Although previous studies in salmon (*Salmo salar*) and medaka have investigated the distribution of *npyb* expression in the brain,^33^, ^46^ they either did not distinguish between sexes or focused solely on females. Thus, to our knowledge, this is the first report of sex differences in *npyb* expression in the teleost brain. Importantly, our finding that *npyb* expression in the Vs/Vp is estrogen‐dependent seems inconsistent with the apparent lack of estrogenic regulation of *npyb* reported in a previous study.^33^ This discrepancy likely reflects the difference in experimental resolution: their whole‐brain real‐time PCR analysis contrasts with our region‐specific in situ hybridization. Because *npyb* is also expressed in the PMp and, more abundantly, in the gc, region‐specific changes in the Vs/Vp may be masked in whole‐brain analysis. Although not statistically significant, their data showed a trend indicating possible estrogen‐dependent regulation of *npyb* expression. While we showed that estrogens can act on *npyb*‐expressing neurons in the Vs/Vp via Esr2b, our transcriptional assay, which included 3 kb upstream and downstream of *npyb*, did not provide evidence for direct transcriptional activation. These results raise the possibility that estrogens regulate *npyb* indirectly through other target genes. Alternatively, estrogens may still directly activate *npyb* transcription via more distal EREs not included in our assay.

Estrogen‐dependent regulation of NPY has also been documented in rodents; however, both the direction of regulation and the specific brain region involved differ from those in medaka. In rodents, NPY expression in the arcuate nucleus is suppressed by ovarian estrogens, in contrast to the estrogen‐induced upregulation of *npyb* in the Vs/Vp of medaka.^47^, ^48^ These estrogenic effects in rodents likely involve membrane‐associated estrogen receptors.^49^ In addition, rodent NPY expression is enhanced by androgens and shows a male‐biased pattern in several brain regions, including the arcuate nucleus, striatum, and hippocampus,^50^ opposite to the case in medaka. These contrasting steroidal effects point to species‐specific modes of NPY regulation in the brain.

NPY is best known for its role in regulating food intake and energy homeostasis. This role appears to be conserved in teleosts, as exogenous NPY alters food intake across several species.^51^, ^52^ Nonetheless, medaka lacking either or both *npya* and *npyb* exhibited normal body growth, comparable to that of wild‐type siblings. Although tangential to the main focus of this study, this observation is noteworthy. These findings echo previous reports in mice and zebrafish, where deletion of NPY does not lead to overt changes in food intake or growth,^41^, ^53^ suggesting robust compensatory mechanisms during development. Indeed, ablation of arcuate NPY neurons in neonatal mice is ineffective, whereas the same manipulation in adults results in drastic reductions in food intake and body weight.^54^ It is thus conceivable that, as in mice, targeting *npya*‐ and/or *npyb*‐expressing neurons in adult medaka could reveal their role. Alternatively, NPY may not play a central role in feeding regulation in medaka. Recent evidence points to agouti‐related peptide (AgRP) as a more critical factor, as *agrp1*‐deficient medaka exhibit reduced food intake and body weight.^33^ Notably, however, deletion of NPY receptors, rather than the ligands, results in growth defects in medaka. Six NPY receptor genes have been identified in this species: *npy2r*, *npy2‐2r*, *npy4r*, *npy7r*, *npy8ar*, and *npy8br*.^55^, ^56^, ^57^ Among these, *npy2r* and *npy8br* mutants have been analyzed, and both showed impaired somatic growth, although only heterozygous mutants were available for *npy2r*.^58^, ^59^ This pattern mirrors findings in mice, where deletion of NPY receptors, but not the ligand, leads to reduced growth.^60^ The physiological basis for this discrepancy between ligand and receptor gene deletions remains elusive and warrants further investigation.

Another noteworthy observation is that, although males lacking either or both *npya* and *npyb* showed no detectable changes in gonadal parameters, females deficient in both genes exhibited a significant reduction in the gonad/body weight ratio. Given that NPY regulates the hypothalamus–pituitary–gonadal axis in female rodents,^61^, ^62^ a similar mechanism may exist in medaka. However, the unchanged gonadal weight, along with previous findings in *Npy*‐deficient mice showing normal ovarian morphology and circulating steroid levels,^63^ suggest that *npya* and *npyb* have, if any, only a minimal role in ovarian development.

In summary, our findings demonstrate that *npyb* expression in the Vs/Vp of medaka is under the control of estrogen/Esr2b signaling and is markedly female‐biased. While the specific molecular link to the behavioral phenotype remains to be fully elucidated, this precise characterization of a major estrogen‐dependent pathway refines the molecular framework downstream of Esr2b and establishes a solid foundation for identifying the mechanisms underlying female mating behavior.

## AUTHOR CONTRIBUTIONS

**Yuki Homan:** Conceptualization; investigation; formal analysis; writing – original draft; funding acquisition. **Yuji Nishiike:** Investigation; formal analysis; writing – review and editing. **Mika Sato‐Tsukamoto:** Investigation; formal analysis; writing – review and editing. **Daisuke Saito:** Formal analysis; writing – review and editing. **Mikita Suyama:** Formal analysis; writing – review and editing. **Yasuyuki Ohkawa:** Formal analysis; writing – review and editing. **Fumiya Takahashi:** Investigation; formal analysis; writing – review and editing. **Takashi Baba:** Investigation; formal analysis; writing – review and editing. **Ken‐ichirou Morohashi:** Formal analysis; writing – review and editing. **Kataaki Okubo:** Conceptualization; formal analysis; supervision; writing – original draft; funding acquisition.

## FUNDING INFORMATION

This work was supported by the Japan Society for the Promotion of Science (JSPS) (grant numbers 23K26998 and 26K01822 to KO). YH was supported by a Sunbor Scholarship from the Suntory Foundation for Life Sciences.

## CONFLICT OF INTEREST STATEMENT

The authors declare no conflicts of interest.

## ETHICS STATEMENT

All animal procedures were conducted in accordance with the guidelines of the University of Tokyo Institutional Animal Care and Use Committee. The committee requests the submission of an animal‐use protocol only for use of mammals, birds, and reptiles, in accordance with the Fundamental Guidelines for Proper Conduct of Animal Experiment and Related Activities in Academic Research Institutions under the jurisdiction of the Ministry of Education, Culture, Sports, Science and Technology of Japan (Ministry of Education, Culture, Sports, Science and Technology, Notice No. 71; June 1, 2006). Accordingly, we did not submit an animal‐use protocol for this study, which used only teleost fish and thus did not require approval by the committee.

## Supporting information


**Table S1.** Species names and GenBank accession numbers of the protein sequences used in this study.
**Table S2.** Primers and a probe used in this study.
**Table S3.** Differentially expressed genes identified through RNA‐seq analysis.
**Table S4.** Abbreviations of medaka brain regions and nuclei.
**Figure S1.** Examination of the potential for estrogen signaling to directly activate *npyb* transcription. (A) Schematic of the *npyb* locus showing the location of canonical bipartite estrogen‐responsive element (ERE)‐like sequences. Bent arrow indicates the transcription start site. Nucleotides of the ERE‐like sequences are shown in uppercase letters, with bases identical to the consensus ERE (AGGTCAnnnTGACCT) highlighted in gray. (B) No detectable induction of *npyb* transcription by estradiol‐17β (E2). Cultured cells were transfected with a luciferase reporter construct containing genomic fragments upstream of the first methionine codon and downstream of the stop codon of *npyb*, along with an expression vector encoding Esr1, Esr2a, or Esr2b. Luciferase activity was measured following treatment with varying E2 concentrations (*n* = 3 per concentration). Statistical significance was evaluated using Dunnett's post hoc test following ANOVA (B). Error bars represent SEM.
**Figure S2.** No detectable deficits in mating behavior, growth, or maturation in *npyb* mutants (Δ29 line). (A)–(D) Body length (A), body weight (B), gonadal weight (C), and gonad/body weight ratio (D) of *npyb* ^+/+^ and *npyb* ^−/−^ females and males of the Δ29 line (*n* = 12 per sex per genotype except for *npyb* ^+/+^ males, where *n* = 11). (E) Latency of *npyb* ^+/+^ and *npyb* ^−/−^ females of the Δ29 line (*n* = 12 per genotype) to receive each mating act (following, courtship display, and wrapping) from the stimulus male and to spawn. (F) Number of courtship displays these females received from the stimulus male and wrapping attempts they rejected. (G) Latency of *npyb* ^+/+^ and *npyb* ^−/−^ males of the Δ29 line (*n* = 12 per genotype) to initiate each mating act toward the stimulus female and to spawn. (H) Number of courtship displays performed by these males and wrapping attempts rejected by the stimulus female. Statistical significance was evaluated using unpaired *t*‐test, with Welch's correction where appropriate (A)–(D), (F), (H), and Gehan–Breslow–Wilcoxon test (E), (G). Error bars represent SEM.
**Figure S3.** Spatial distribution of *pyya* and *pyyb* expression in the brain. (A) Phylogenetic tree illustrating the relationship of medaka Pyya and Pyyb to other PYY homologs. Numbers at nodes indicate bootstrap values from 1000 replicates. Scale bar: 0.1 substitutions per site. (B) Lateral schematic of the medaka brain (anterior to the left) indicating section planes shown in panel (C). (C) Coronal brain sections highlighting nuclei with detectable *pyya* and *pyyb* expression (indicated by stars). (D), (E) Representative images of *pyya* (D) and *pyyb* (E) expression in each nucleus of wild‐type females and males. (F), (G) Relative expression levels of *pyya* (F) and *pyyb* (G) in each brain region of *esr2b* ^+/+^ and *esr2b* ^−/−^ females and males, measured by real‐time PCR (*n* = 5–6 per sex per genotype). Species names and GenBank accession numbers are provided in Table S1. Abbreviations of brain regions and nuclei are given in Table S4. Statistical significance was evaluated using Bonferroni's post hoc test following ANOVA (F, G).
**Figure S4.** No detectable deficits in mating behavior, growth, or maturation in *npya* mutants (Δ50 line). (A) Schematic of the *npya* locus showing the CRISPR RNA target site, which is enlarged to depict the nucleotide sequences of the wild‐type (+) and mutant (Δ50 and Δ53) alleles. The CRISPR RNA target sequence is underlined, and deleted bases are indicated by dashes. (B) Comparison of the deduced precursor proteins of wild‐type (+) and mutant (Δ50 and Δ53) Npya. The mature Npya polypeptide is boxed. Altered sequences resulting from frameshifts are shaded gray. Asterisks denote stop codons. (C) Relative transcript levels of *npya* in the brains of *npya* ^+/+^ and *npya* ^−/−^ females of the Δ53 line, measured by real‐time PCR (*n* = 5–6 per genotype). (D–G) Body length (D), body weight (E), gonadal weight (F), and gonad/body weight ratio (G) of *npya* ^+/+^ and *npya* ^−/−^ females and males of the Δ50 line (*n* = 12 per sex per genotype except for *npya* ^+/+^ males, where *n* = 11). (H) Latency of *npya* ^+/+^ and *npya* ^−/−^ females of the Δ50 line (*n* = 12 and 10, respectively) to receive each mating act (following, courtship display, and wrapping) from the stimulus male and to spawn. (I) Number of courtship displays these females received from the stimulus male and wrapping attempts they rejected. (J) Latency of *npya* ^+/+^ and *npya* ^−/−^ males of the Δ50 line (*n* = 12 per genotype) to initiate each mating act toward the stimulus female and to spawn. (K) Number of courtship displays performed by these males and wrapping attempts rejected by the stimulus female. Statistical significance was evaluated using unpaired *t*‐test, with Welch's correction where appropriate (C)–(G), (I), (K), and Gehan–Breslow–Wilcoxon test (H), (J). Error bars represent SEM.
**Figure S5.** No detectable deficits in mating behavior, growth, or maturation in *npya* mutants (Δ53 line). (A)–(D) Body length (A), body weight (B), gonadal weight (C), and gonad/body weight ratio (D) of *npya* ^+/+^ and *npya* ^−/−^ females and males of the Δ53 line (*n* = 12 per sex per genotype except for *npya* ^+/+^ females, where *n* = 10). (E) Latency of *npya* ^+/+^ and *npya* ^−/−^ females of the Δ53 line (*n* = 12 per genotype) to receive each mating act (following, courtship display, and wrapping) from the stimulus male and to spawn. (F) Number of courtship displays these females received from the stimulus male and wrapping attempts they rejected. (G) Latency of *npya* ^+/+^ and *npya* ^−/−^ males of the Δ53 line (*n* = 12 per genotype) to initiate each mating act toward the stimulus female and to spawn. (H) Number of courtship displays performed by these males and wrapping attempts rejected by the stimulus female. Statistical significance was evaluated using unpaired *t*‐test, with Welch's correction where appropriate (A)–(D), (F), (H), and Gehan–Breslow–Wilcoxon test (E), (G). Error bars represent SEM.
**Figure S6.** Assessment of potential compensatory expression changes in NPY/PYY family members in NPY‐deficient females by real‐time PCR. (A) Relative expression levels of *npya*, *pyya*, and *pyyb* in the brains of *npyb* ^+/+^ and *npyb* ^−/−^ females of the Δ5 line (*n* = 5–6 per genotype). (B) Relative expression levels of *npyb*, *pyya*, and *pyyb* in the brains of *npya* ^+/+^ and *npya* ^−/−^ females of the Δ53 line (*n* = 5–6 per genotype). (C) Relative expression levels of *pyya* and *pyyb* in the brains of *npya* ^+/−^/*npyb* ^+/−^ and *npya* ^−/−^/*npyb* ^−/−^ females (*n* = 6 per genotype). Statistical significance was evaluated using unpaired *t*‐test (A)–(C). Error bars represent SEM.

## Data Availability

The RNA‐seq data have been deposited in the DDBJ Sequenced Read Archive under the accession numbers DRR749573 to DRR749604. All other data supporting the findings of this study are available within the article and its supplementary information.
