# Supplementary material for: Estrogen/Esr2b signaling‐dependent, female‐biased expression of a neuropeptide Y gene in the medaka brain
Source: J Neuroendocrinol. 2026 Sep 30;38(10):e70279. doi: 10.1111/jne.70279 (PMC13624830; doi:10.1111/jne.70279)
Supplement: Supplementary file 1 [file JNE-38-e70279-s001.docx]

**Table S1. Species names and GenBank accession numbers of the protein sequences used in this study.**

| protein | species | accession number |
| --- | --- | --- |
| NPY | human (*Homo sapiens*) | NP_000896 |
| NPY | mouse (*Mus musculus*) | NP_075945 |
| Npy | chicken (*Gallus gallus*) | NP_990804 |
| Npy | *Xenopus tropicalis* | NP_001072530 |
| Npya (predicted) | stickleback (*Pungitius pungitius*) | XP_037330437 |
| Npya (predicted) | medaka (*Oryzias latipes*) | XP_011478948 |
| Npya | zebrafish (*Danio rerio*) | NP_571149 |
| Npyb (predicted) | stickleback (*Pungitius pungitius*) | XP_037308919 |
| Npyb (predicted) | medaka (*Oryzias latipes*) | XP_020565797 |
| PYY | human (*Homo sapiens*) | NP_001380957 |
| PYY | mouse (*Mus musculus*) | NP_663410 |
| Pyy | *Xenopus tropicalis* | NP_001072427 |
| Pyya (predicted) | stickleback (*Gasterosteus aculeatus*) | XP_040047889 |
| Pyya (predicted) | medaka (*Oryzias latipes*) | XP_023813654 |
| Pyya | zebrafish (*Danio rerio*) | NP_571091 |
| Pyyb (predicted) | stickleback (*Gasterosteus aculeatus*) | XP_040032727 |
| Pyyb (predicted) | medaka (*Oryzias latipes*) | XP_023805359 |
| Pyyb | zebrafish (*Danio rerio*) | NP_001314824 |

**Table S2. Primers and a probe used in this study.**

|  | target | direction | purpose | sequence (5′–3′) |
| --- | --- | --- | --- | --- |
| primer | *npyb* | forward | real-time PCR | CCGGGCTCCAACTATATCAGG |
| primer | *npyb* | reverse | real-time PCR | GGGTAATGTAAAATCACTGGCAGA |
| primer | *npyb* | forward | genotyping (gDNA PCR) | ATAAAGATGGCCAGGGTTGGGA |
| primer | *npyb* | reverse | genotyping (gDNA PCR) | GCAAAGTCTGCTGCCTCAAATG |
| primer | *npyb* | forward | genotyping (CS) | ACTTAGTAGATCTGCTACAGTCCA |
| primer | *npyb* | forward | genotyping (HRM) | CTCTCTGCCTGCTCCTCTGT |
| primer | *npyb* | reverse | genotyping (HRM) | AGATTGATATAGTGTCGCAGTGCA |
| probe | *npyb* | forward | genotyping (HRM) | AAGCCTCCTGGTCCCCGGGAAGGAGCG |
| primer | *npya* | forward | real-time PCR | GAAAGCAAAGACACACTTCCACA |
| primer | *npya* | reverse | real-time PCR | AAGGCATGAGGGTAGAGTGAGG |
| primer | *npya* | forward | genotyping (gDNA PCR) | ATGACTGAAATGAAGGAGACCAAG |
| primer | *npya* | reverse | genotyping (gDNA PCR) | CATTCAGAGAGAAGCTCAAAACTG |
| primer | *npya* | reverse | genotyping (CS) | ATAAGCCATCACGGTCACTTAATC |
| primer | *pyya* | forward | real-time PCR | ATCTTCCTGTGTGTGCTCGTGT |
| primer | *pyya* | reverse | real-time PCR | TGTAGTACTTGGCCAGCTCTTCG |
| primer | *pyyb* | forward | real-time PCR | TATGTCAACCTCATCACCAGGC |
| primer | *pyyb* | reverse | real-time PCR | GAGGCTCTGCATCTTGACTTGA |
| primer | *actb* | forward | real-time PCR | CCCCACCCAAAGTTTAG |
| primer | *actb* | reverse | real-time PCR | CAACGATGGAGGGAAAGACA |

gDNA PCR, PCR on genomic DNA; CS, cycle sequence; HRM, high-resolution melting analysis.

**Table S3. Differentially expressed genes identified through RNA-seq analysis.**

| gene ID | gene symbol (description) | GenBank accession number | FDR in Tel+OB | FDR in MT+Die | FDR in OT+OpN | FDR in Cb+MO |
| --- | --- | --- | --- | --- | --- | --- |
| XLOC_007083 | si:dkey-103j14.5 (isoaspartyl peptidase/L-asparaginase) | XM_004077202 | 0.221 | 0.049 * | 0.614 | 1.000 |
| XLOC_008613 | *npyb* (neuropeptide Yb) | XM_020710138 | 2.71×10^-^¹¹ *** | 0.921 | 0.857 | 1.000 |
| XLOC_009226 | *cahz* (carbonic anhydrase) | XM_023964792 | 0.034 * | 0.988 | 0.673 | 1.000 |
| XLOC_009657 | *blvra* (biliverdin reductase A) | XM_004078625 | 0.554 | 0.490 | 0.042 * | 1.000 |
| XLOC_011292 | LOC101171201 (hemoglobin subunit α1-like) | NM_001278798 | 0.048 * | 0.980 | 0.655 | 1.000 |
| XLOC_019661 | *mibp* (muscle-specific beta 1 integrin binding protein) | XM_004085846 | 0.026 * | 0.958 | 0.398 | 1.000 |
| XLOC_022036 | *ldha* (lactate dehydrogenase A4) | XM_023956195 | 0.982 | 0.983 | 0.008 ** | 1.000 |
| XLOC_024194 | LOC101161891 (testis-expressed protein 2-like) | XM_004071689 | 0.039 * | 0.999 | 0.904 | 1.000 |
| XLOC_024417 | *epor* (erythropoietin receptor) | XM_004071990 | 0.022 * | 0.947 | 0.614 | 1.000 |
| XLOC_024749 | LOC101174997 (hemoglobin embryonic subunit α-like) | XM_004071343 | 0.034 * | 0.972 | 0.672 | 1.000 |
| XLOC_024810 | *npba* (neuropeptide Ba) | NM_001308979 | 3.36×10^-^¹⁵ *** | 0.813 | 0.816 | 1.000 |
| XLOC_025394 | *rmi1* (RecQ mediated genome instability 1) | XM_023958384 | 0.020 * | 0.711 | 0.439 | 0.058 |
| XLOC_025974 | *slc23a2* (solute carrier family 23 member 2) | XM_004072276 | 0.034 * | 0.400 | 0.124 | 0.087 |
| XLOC_025983 | LOC111947880 (prostate stem cell antigen-like) | XM_023958284 | 0.318 | 0.318 | 0.350 | 0.040 * |

FDR: false discovery rate. *FDR < 0.05, **FDR < 0.01, ***FDR < 0.001.

**Table S4. Abbreviations of medaka brain regions and nuclei.**

| abbreviation | full name | location |
| --- | --- | --- |
| brain region |  |  |
| Cb | cerebellum |  |
| Die | diencephalon |  |
| MO | medulla oblongata |  |
| MT | midbrain tegmentum |  |
| OB | olfactory bulb |  |
| OpN | optic nerve |  |
| OT | optic tectum |  |
| Tel | telencephalon |  |
| brain nucleus |  |  |
| Dl | lateral nucleus of the dorsal telencephalic area | dorsal telencephalon |
| Dm | medial nucleus of the dorsal telencephalic area | dorsal telencephalon |
| DT | dorsal tegmental nucleus | midbrain tegmentum |
| Flt | telencephalic lateral longitudinal fascicle | ventral telencephalon |
| gc | central gray | brainstem |
| NAT | anterior tuberal nucleus | hypothalamus |
| NPPv | posterior periventricular nucleus | hypothalamus |
| NRL | lateral recess nucleus | hypothalamus |
| NVT | anterior part of the ventral tuberal nucleus | hypothalamus |
| Pbl | basal lateral preoptic nucleus | preoptic area |
| PGZ3 | periventricular gray zone (layer 3) | optic tectum |
| PMp | parvocellular portion of the magnocellular preoptic nucleus | preoptic area |
| Vl | lateral nucleus of the ventral telencephalic area | ventral telencephalon |
| VM | ventromedial nucleus | thalamus |
| Vp | posterior nucleus of the ventral telencephalic area | ventral telencephalon |
| Vs | supracommissural nucleus of the ventral telencephalic area | ventral telencephalon |

**Figure S1. Examination of the potential for estrogen signaling to directly activate *npyb* transcription.** (A) Schematic of the *npyb* locus showing the location of canonical bipartite estrogen-responsive element (ERE)-like sequences. Bent arrow indicates the transcription start site. Nucleotides of the ERE-like sequences are shown in uppercase letters, with bases identical to the consensus ERE (AGGTCAnnnTGACCT) highlighted in gray. (B) No detectable induction of *npyb* transcription by estradiol-17β (E2). Cultured cells were transfected with a luciferase reporter construct containing genomic fragments upstream of the first methionine codon and downstream of the stop codon of *npyb*, along with an expression vector encoding Esr1, Esr2a, or Esr2b. Luciferase activity was measured following treatment with varying E2 concentrations (n = 3 per concentration). Statistical significance was evaluated using Dunnett’s post hoc test following ANOVA (B). Error bars represent SEM.

**Figure S2. No detectable deficits in mating behavior, growth, or maturation in *npyb* mutants (Δ29 line).** (A–D) Body length (A), body weight (B), gonadal weight (C), and gonad/body weight ratio (D) of *npyb*^+/+^ and *npyb*^−/−^ females and males of the Δ29 line (n = 12 per sex per genotype except for *npyb*^+/+^ males, where n = 11). (E) Latency of *npyb*^+/+^ and *npyb*^−/−^ females of the Δ29 line (n = 12 per genotype) to receive each mating act (following, courtship display, and wrapping) from the stimulus male and to spawn. (F) Number of courtship displays these females received from the stimulus male and wrapping attempts they rejected. (G) Latency of *npyb*^+/+^ and *npyb*^−/−^ males of the Δ29 line (n = 12 per genotype) to initiate each mating act toward the stimulus female and to spawn. (H) Number of courtship displays performed by these males and wrapping attempts rejected by the stimulus female. Statistical significance was evaluated using unpaired t test, with Welch’s correction where appropriate (A–D, F, H), and Gehan–Breslow–Wilcoxon test (E, G). Error bars represent SEM.

**Figure S3. Spatial distribution of *pyya* and *pyyb* expression in the brain.** (A) Phylogenetic tree illustrating the relationship of medaka Pyya and Pyyb to other PYY homologs. Numbers at nodes indicate bootstrap values from 1000 replicates. Scale bar: 0.1 substitutions per site. (B) Lateral schematic of the medaka brain (anterior to the left) indicating section planes shown in panel C. (C) Coronal brain sections highlighting nuclei with detectable *pyya* and *pyyb* expression (indicated by stars). (D, E) Representative images of *pyya* (D) and *pyyb* (E) expression in each nucleus of wild-type females and males. (F, G) Relative expression levels of *pyya* (F) and *pyyb* (G) in each brain region of *esr2b*^+/+^ and *esr2b*^−/−^ females and males, measured by real-time PCR (n = 5–6 per sex per genotype). Species names and GenBank accession numbers are provided in Table S1. Abbreviations of brain regions and nuclei are given in Table S4. Statistical significance was evaluated using Bonferroni’s post hoc test following ANOVA (F, G).

**Figure S4. No detectable deficits in mating behavior, growth, or maturation in *npya* mutants (Δ50 line).** (A) Schematic of the *npya* locus showing the CRISPR RNA target site, which is enlarged to depict the nucleotide sequences of the wild-type (+) and mutant (Δ50 and Δ53) alleles. The CRISPR RNA target sequence is underlined, and deleted bases are indicated by dashes. (B) Comparison of the deduced precursor proteins of wild-type (+) and mutant (Δ50 and Δ53) Npya. The mature Npya polypeptide is boxed. Altered sequences resulting from frameshifts are shaded gray. Asterisks denote stop codons. (C) Relative transcript levels of *npya* in the brains of *npya*^+/+^ and *npya*^−/−^ females of the Δ53 line, measured by real-time PCR (n = 5–6 per genotype). (D–G) Body length (D), body weight (E), gonadal weight (F), and gonad/body weight ratio (G) of *npya*^+/+^ and *npya*^−/−^ females and males of the Δ50 line (n = 12 per sex per genotype except for *npya*^+/+^ males, where n = 11). (H) Latency of *npya*^+/+^ and *npya*^−/−^ females of the Δ50 line (n = 12 and 10, respectively) to receive each mating act (following, courtship display, and wrapping) from the stimulus male and to spawn. (I) Number of courtship displays these females received from the stimulus male and wrapping attempts they rejected. (J) Latency of *npya*^+/+^ and *npya*^−/−^ males of the Δ50 line (n = 12 per genotype) to initiate each mating act toward the stimulus female and to spawn. (K) Number of courtship displays performed by these males and wrapping attempts rejected by the stimulus female. Statistical significance was evaluated using unpaired t test, with Welch’s correction where appropriate (C–G, I, K), and Gehan–Breslow–Wilcoxon test (H, J). Error bars represent SEM.

**Figure S5. No detectable deficits in mating behavior, growth, or maturation in *npya* mutants (Δ53 line).** (A–D) Body length (A), body weight (B), gonadal weight (C), and gonad/body weight ratio (D) of *npya*^+/+^ and *npya*^−/−^ females and males of the Δ53 line (n = 12 per sex per genotype except for *npya*^+/+^ females, where n = 10). (E) Latency of *npya*^+/+^ and *npya*^−/−^ females of the Δ53 line (n = 12 per genotype) to receive each mating act (following, courtship display, and wrapping) from the stimulus male and to spawn. (F) Number of courtship displays these females received from the stimulus male and wrapping attempts they rejected. (G) Latency of *npya*^+/+^ and *npya*^−/−^ males of the Δ53 line (n = 12 per genotype) to initiate each mating act toward the stimulus female and to spawn. (H) Number of courtship displays performed by these males and wrapping attempts rejected by the stimulus female. Statistical significance was evaluated using unpaired t test, with Welch’s correction where appropriate (A–D, F, H), and Gehan–Breslow–Wilcoxon test (E, G). Error bars represent SEM.

**Figure S6. Assessment of potential compensatory expression changes in NPY/PYY family members in NPY-deficient females by real-time PCR.** (A) Relative expression levels of *npya*, *pyya*, and *pyyb* in the brains of *npyb*^+/+^ and *npyb*^−/−^ females of the Δ5 line (n = 5–6 per genotype). (B) Relative expression levels of *npyb*, *pyya*, and *pyyb* in the brains of *npya*^+/+^ and *npya*^−/−^ females of the Δ53 line (n = 5–6 per genotype). (C) Relative expression levels of *pyya* and *pyyb* in the brains of *npya*^+/−^/*npyb*^+/−^ and *npya*^−/−^/*npyb*^−/−^ females (n = 6 per genotype). Statistical significance was evaluated using unpaired t test (A–C). Error bars represent SEM.
